# General Anesthesia and the Young Brain: The Importance of Novel Strategies with Alternate Mechanisms of Action

**DOI:** 10.3390/ijms23031889

**Published:** 2022-02-08

**Authors:** Stefan Maksimovic, Nemanja Useinovic, Nidia Quillinan, Douglas F. Covey, Slobodan M. Todorovic, Vesna Jevtovic-Todorovic

**Affiliations:** 1 Department of Anesthesiology, Anschutz Medical Campus, University of Colorado, Aurora, CO 80045, USA; nemanja.useinovic@cuanschutz.edu (N.U.); nidia.quillinan@cuanschutz.edu (N.Q.); slobodan.todorovic@cuanschutz.edu (S.M.T.); vesna.jevtovic-todorovic@cuanschutz.edu (V.J.-T.); 2 Neuronal Injury and Plasticity Program, Anschutz Medical Campus, University of Colorado, Aurora, CO 80045, USA; 3 Department of Developmental Biology, Washington University in St. Louis School of Medicine, St. Louis, MO 63110, USA; dcovey@wustl.edu; 4 Taylor Family Institute for Innovative Psychiatric Research, Washington University in St. Louis School of Medicine, St. Louis, MO 63110, USA; 5 Department of Pharmacology, Anschutz Medical Campus, University of Colorado, Aurora, CO 80045, USA

**Keywords:** general anesthetics, neurotoxicity, neuroactive steroid analogues, synaptogenesis

## Abstract

Over the past three decades, we have been grappling with rapidly accumulating evidence that general anesthetics (GAs) may not be as innocuous for the young brain as we previously believed. The growing realization comes from hundreds of animal studies in numerous species, from nematodes to higher mammals. These studies argue that early exposure to commonly used GAs causes widespread apoptotic neurodegeneration in brain regions critical to cognition and socio-emotional development, kills a substantial number of neurons in the young brain, and, importantly, results in lasting disturbances in neuronal synaptic communication within the remaining neuronal networks. Notably, these outcomes are often associated with long-term impairments in multiple cognitive-affective domains. Not only do preclinical studies clearly demonstrate GA-induced neurotoxicity when the exposures occur in early life, but there is a growing body of clinical literature reporting similar cognitive-affective abnormalities in young children who require GAs. The need to consider alternative GAs led us to focus on synthetic neuroactive steroid analogues that have emerged as effective hypnotics, and analgesics that are apparently devoid of neurotoxic effects and long-term cognitive impairments. This would suggest that certain steroid analogues with different cellular targets and mechanisms of action may be safe alternatives to currently used GAs. Herein we summarize our current knowledge of neuroactive steroids as promising novel GAs.

## 1. General Anesthesia and Neurodevelopment

The last three decades of extensive research have unveiled concerning effects of general anesthetics on the developing brain. Described in different species (from nematodes to nonhuman primates) those effects range from neuronal and glial damage [1,2,3,4,5,6,7,8] and functional impairments in neuronal communications to long-lasting socioaffective and cognitive deficits [8,9,10,11,12,13,14]. Considering that the majority of currently used general anesthetics are known to be detrimental to a very young brain, the pressing concern has been centered around the development of safe general anesthetics that lack neurotoxic properties.

Based on numerous preclinical studies, it appears that the mammalian brain is most vulnerable during the intense period of synaptogenesis, a stage in which trillions of neuronal synapses are being formed and the neurons actively migrate to reach their final destination while connecting and maturing, thus forming a complex and robust network often referred to as neuronal circuitries, which are crucially important for proper behavioral and cognitive development [1,2,3,4,5,6,7,8]. It is important to note that synaptogenesis relies on timely maturation and proliferation of glial cells that provide a nurturing environment, thus setting the stage for developmental synaptogenesis. The rodent species (mice and rats) have been most often used in currently published preclinical studies. Their synaptogenesis is mainly a postnatal phenomenon lasting about three weeks, with the peak occurring around the postnatal day 7, which is repeatedly reported to be most susceptible time period when it comes to general-anesthesia-induced developmental neurotoxicity [15]. It is important to notice that synaptogenesis in humans, although variable across the brain regions, begins in the fetal period, reaching the maximum by the second year of life postnatally [16].

Significant neuronal demise during this critical stage has been shown to result in long-lasting disturbances in neuronal synaptic communications within the remaining neuronal networks believed to be, at least in part, the functional read-out of the deficits in cognitive, affective, and motor behaviors detected later in life [9,10,11,12,13,14,17,18,19]. Although not surprising when considering ever-mounting animal evidence, more concerning is the steadily growing body of clinical evidence suggesting that early exposure to general anesthetics appears to have deleterious effects on behavioral performance during adolescence (e.g., learning disabilities, attention deficit disorders, poor school performance, socioaffective impairments, etc.) [14,17,18,19]. To keep the public informed, a few years ago the U.S. Food and Drug Administration issued a Drug and Safety Communication statement about the potential risk for neurodevelopmental impairments in children prenatally or postnatally exposed to repeated or prolonged general anesthesia [20]. Although the need to provide comfort during painful procedures is beyond dispute, the struggle to reassure that we do no harm to our youngest patient population during critical stages of their brain development remains at the forefront of our efforts.

As stated earlier, general-anesthesia-induced impairment of developmental synaptogenesis is marked by massive and widespread neuroapoptosis, as reported in several mammalian species (e.g., mice, rats, guinea pigs, piglets, and monkeys). Due to their unique role in promoting all aspects of synaptogenesis and culpable role in initiating the cascade of apoptotic events ultimately leading to neuronal death (via the intrinsic pathway activation), mitochondria became a primary target of interest. By downregulating antiapoptotic proteins from the bcl-2 family (e.g., bcl-x_L_), which protect mitochondrial integrity, general anesthetics promote an increase in mitochondrial membrane permeability, followed by increased cytochrome c release into the cytoplasm, which in turn activates caspase-9 and -3, resulting in apoptosis [2,3,21,22,23]. In addition to this fairly acute process, general anesthetics cause a significant and long-lasting disturbance in mitochondrial morphogenesis. Two weeks after anesthesia exposure, mitochondrial enlargement and deranged, fragmented cristae and inner membranes were reported, suggesting significant impairment of mitochondrial membrane integrity and deranged mitochondrial dynamics indicative of imbalance between fission and fusion [21,22]. It appears that general anesthetics favor excessive fusion by affecting homeostasis of two important GTPase proteins—Drp1 and mitofusin 2—which are critical for proper fusion and fission pathway activation and mitochondrial regeneration. It is understandable that this disbalance in mitochondrial dynamics may not be well tolerated by immature and functionally engaged mammalian neurons that need adequate metabolic support. Of particular interest for the reported functional impairments is the fact that although mitochondria are generated in the neuronal body, they need to traverse the dendrites and axons to position themselves strategically at the vicinity of active growth cones and in terminals with active synapses to ensure adequate ATP production. Our recent work suggested that significantly fewer mitochondria are located in presynaptic neuronal profiles in the general-anesthesia-treated brain compared to controls [23]. Since these mitochondria also are significantly larger than those in controls, it was proposed that anesthesia-induced mitochondrial enlargement causes mitochondria to be sluggish and ‘stuck’ in more proximal cellular compartments, thus shifting their regional distribution away from very distant thin and highly arborized dendritic branches at a time when their presence is necessary for normal synapse formation and development. In fact, we and others have reported that anesthesia impairs plasticity of dendritic spines and the formation, stability, and function of developing synapses [24,25,26,27,28,29]. Moreover, morphological distortion and impaired regional distribution of mitochondria results in mitochondria-induced reactive oxygen species upregulation, lipid peroxidation, and neuronal deletion, which may contribute to the observed impairment of synapses and neuronal network formation, ultimately leading to functional impairments [30].

### Impairment of Neuronal Networks Caused by an Early Exposure of the Developing Brain to General Anesthesia 

Although our initial efforts in the field of developmental neurotoxicity were focused on examining the nature of the morphological changes that can be easily detected using histological assessments, we learned that the general-anesthesia-induced impairment of synaptogenesis is multifaceted, and that seemingly subtle changes that cannot be detected morphologically remain in surviving ‘normal’ neurons after the grossly damaged neurons have been removed. Based on presently available evidence, these neurons may not be truly functional; i.e., their communications may be faulty.

We first noted that an early exposure to general anesthesia causes long-term impairment in synaptic transmission in the hippocampus of adolescent rats (postnatal days 27–33) exposed to anesthesia at the peak of their synaptogenesis (postnatal day 7) [2]. In particular, long-term potentiation was impaired significantly despite the presence of robust short-term potentiation. This observation suggested a long-lasting disturbance in neuronal circuitries in the young hippocampus, a brain region that is crucial for proper learning and memory development. A deficit in long-term potentiation was confirmed when synaptic transmission was examined using patch-clamp recordings of evoked inhibitory postsynaptic current (eIPSC) and evoked excitatory postsynaptic current (eEPSC) by recording from the pyramidal layer of control and anesthesia-treated rat subiculum, an important component of the hippocampal complex. Again, it was noted that anesthesia-treated animals suffered from impaired synaptic transmission, with inhibitory transmission affected significantly [24].

The general anesthetics commonly used in clinical settings are known to modulate two main neurotransmitter systems in the developing brain: γ-aminobutyric acid (GABA) and N-methyl-D-aspartate (NMDA) [31]. Historically, general anesthetics were considered ‘muddy players’ regarding their ability to interfere with the milieu of receptors present in the brain. Because of their promiscuity, they have high potency to transiently inhibit communication in the central nervous system. Putatively, this inhibition is maintained by blocking glutamatergic NMDA receptors and/or potentiating chloride influx through interaction with GABA receptors. Whilst the first insight into excessive excitation via NMDA receptors revealed increased cell death, later findings confirmed that inhibition of glutamate influx through these receptors was equally detrimental. This phenomenon was observed after the application of a commonly used intravenous general anesthetic—ketamine [32]. Moreover, propofol together with most volatile agents (e.g., sevoflurane, isoflurane, etc.) increases the sensitivity of GABA receptors, therefore potentiating the influx of chloride inside the neurons and causing inhibition of further transmission. Although the cellular mechanisms of general anesthetics are not fully understood, there is a preponderance of evidence suggesting that unphysiological modulation of these two neurotransmitter systems results in widespread neurotoxicity during synaptogenesis [1,2,3,4,5,6,7]. Of particular concern is recent work that suggests that these deleterious effects may be transgenerational via epigenetic modulation of the epigenome, thus signifying that the effects of general anesthetics are not only long-lasting but could be embedded in the genome, allowing for transgenerational impairments in offspring never exposed to general anesthetics [33]. Taking this information into consideration, we and others have been putting considerable effort into discovering new molecular targets that could guide the development of novel general anesthetics that are safe for use in the very young.

This review will focus on recent work with novel neuroactive steroids, a class of steroids with activity in the brain that are prepared by chemical synthesis. Recently collected evidence suggests that they could be considered promising and safe general anesthetics devoid of neurotoxic effects in very young brain [34,35], and as such, a promising and safe alternative to common general anesthetics currently used in pediatric practice.

## 2. Neuroactive Steroids as Promising Therapeutic Agents

It is well-accepted that neuroactive steroids, a very heterogenous group of molecules, regulate many physiological functions in the central nervous system, especially during development. Endogenous synthesis of these compounds takes place in the peripheral endocrine glands, but many regions in the brain have the necessary enzymes for the conversion of cholesterol into pregnanolone, which is the first step in steroidogenesis. Their roles in development, as well as in physiological and pathological processes in the brain, raised the question about targeting neuroactive steroids and their metabolism for future therapeutic interventions [36,37,38].

The idea that neuroactive steroids could be favorable therapeutic agents for a vast array of pathological conditions (e.g., postpartum depression, anxiety, etc.) has been considered for quite some time [39]. For example, many previous studies have suggested the neuroprotective potential of neuroactive steroids in pathological conditions such as hypoxia, Alzheimer’s disease, and ischemia, to name a few, with therapeutic potential being linked to their effects on nuclear steroid hormone receptors and control of gene expression [40,41,42]. Furthermore, neuroactive steroids, both endogenous and exogenous, are also known to interact with membrane surface ligand- and voltage-gated ion channels [43,44,45]. In addition to being recognized as sedative agents, some synthetic analogues of endogenous neurosteroids have been under investigation in a couple of preclinical and clinical studies for the treatment of epilepsy [38,46,47]. Interestingly, endogenous neurosteroids have been implicated in chronic stress conditions, whereby their levels are found to be decreased resulting in psychiatric disorders [48,49], as well as hypothalamic–pituitary–adrenal axis dysregulation [50]. It is noteworthy that neuroactive steroids with GABA-modulatory properties seem to have biphasic actions—moderate levels inhibit the GABA_A_ receptors, whereas lower and higher concentrations potentiate GABA-modulatory activity [44,45].

Some steroids involved in the development of the central nervous system are directly synthesized from their precursors in the brain. Namely, these neuroactive steroids, such as 17β-estradiol and dihydrotestosterone, as well other metabolites from testosterone, critically affect different processes such as sexual dimorphism, organizational changes in synapses, neuronal density, reproductive behavior, etc. These effects are exhibited through the interaction with intracellular steroid receptors activating transcription factors, as well as membrane-bound receptors, such as GABA, 5-HT_3_, etc. [37]. Of importance in this review is a report that some of the steroids, such as the enantiomer of 17β-estradiol, may modulate prosurvival signaling pathways that, for example, induce an increase in brain-derived neurotrophic factor (BDNF) levels, which not only improves learning and memory, but importantly, reverses cognitive impairments caused by general anesthetics [13,51,52,53,54].

Allopregnanolone (3α-hydroxy-5α-pregnan-20-one), an endogenous steroid that plays an important role in the development of the mammalian brain in utero, was reported to confer neuroprotection via various membrane-bound progesterone and xenobiotic receptors. In this regard, a recently published report suggested that allopregnanolone, much like 17β-estradiol, protects against ketamine-induced apoptosis in cultured neural stem cells [55]. While these studies suggested neuroactive steroids can be used as neuroprotective agents, recent efforts have revealed their potential as anesthetic/hypnotic agents.

Hence, presently available evidence suggests that neuroactive steroids are complex agents that may exert significant dose- and time-dependent pharmacological effects, and as such should be carefully considered in view of specific circumstances brought about by different pathophysiological conditions.

## 3. Neuroactive Steroids: Endogenous and Synthetic Compounds with Hypnotic Properties but without Neurotoxic Effects

### 3.1. Chemical Structure and Related Functions of Neuroactive Steroids; Insights from Presently Available Structure-Activity Studies

In the mid-20th century, the field of anesthesiology introduced a type of general anesthesia referred to as ‘steroid anesthesia’, based on the use of a variety of synthetic neuroactive steroids with sedative and hypnotic properties [43]. The best-known neuroactive steroids are alphaxolone, alphadolone, hydroxydione, and minaxolone. The first to be introduced was hydroxydione, which is the esterified 21-hydroxy derivative of 5β-pregnanedione. Hydroxydione was a useful general anesthetic with a good safety profile, although it was repeatedly reported to cause pain and irritation at the site of injection, most likely due to poor solubility. Although these first attempts at providing steroid anesthesia were deemed risky due to significant side effects and hence were discontinued, the field has gained valuable experience with their clinical use, and learned that reported complications were mainly due to the pharmaceutical formulations used to dissolve them. For example, one of the analogues, alphaxalone, considered to be the most successful attempt at steroid anesthesia in both human and veterinary practice [56], was reported to cause a higher incidence of anaphylactic reactions. Although it was later determined that the vehicle was the culprit, alphaxalone nonetheless fell out of favor as an anesthetic [56,57,58,59].

The basic structure of a neuroactive steroid consists of four rings with varying reactive functional groups that can be modified to yield different functional properties [60,61]. Nowadays, based on the available literature and progress in this field, we know that the most pronounced sedative and hypnotic effects of neuroactive steroids come from the location of a hydroxyl group in the alpha configuration at C3, and with a hydrogen at C5 in either the alpha or beta configurations. For example, this finding has been exemplified for neurosteroid analogues having a carbonitrile group on carbon 17 in the β configuration [62]. Moreover, in that study, using whole-patch electrophysiological recordings from cultured hippocampal neurons, as well as molecular modeling methods, it was shown that the configuration of the hydroxyl group at C-3 is a more important element with regard to anesthetic potency than the configuration at C-5 [62].

Numerous studies have established that the analogues with strong GABA-modulatory properties are the most potent general anesthetics [44,45,60,61,62,63,64,65]. GABA_A_ receptor, a ligand-gated chloride ion channel, has been traditionally considered the most important target for general anesthetics in hypnotically relevant concentrations. By increasing chloride influx via GABA_A_ receptor activation, membrane hyperpolarization is observed in mature neurons. However, in the immature neurons, GABA receptor activation results in membrane depolarization and an increase in neuronal firing [66,67], a phenomenon that has been studied for decades.

Recently, it was suggested that other cellular targets such as various ligand-gated and voltage-gated ion channels could play an important role in clinical effects of general anesthetics as well [67,68,69]. To this end, a promising cellular target for the development of novel general anesthetics of particular interest for quite some time has been a subtype of voltage-gated calcium channels referred to as T-type calcium channels (T-channels). Known to be activated at low voltage, they are uniquely positioned to regulate many aspects of synaptic neurotransmission and neuronal excitability [68], as well as to modulate pain transmission and seizure activity [68,69,70,71,72]. Consequently, agents that block T-channels have been proposed as novel treatment options for certain conditions such as acute and chronic pain [55,59], as well as seizure disorders [69,70,71,72,73]. T-channels (Ca_v_3) are considered the least homologous subfamily of voltage-gated ion channels, with limited overlap (only about 25% amino acid sequence) with other voltage-gated calcium channels (Ca_v_1 and Ca_v_2). Considering that the density of T-channels at the plasma membranes may be variable, their role at regulating Ca^2+^-activated processes may also be via different signaling pathways, rather than by a global and massive rise in cytosolic Ca^2+^ levels, which directly regulate neuronal excitability [68,71]. Over the past several decades, the field of voltage-gated calcium channels has recognized three distinct members of the Ca_v_3 family first identified at the molecular level: Ca_v_3.1, Ca_v_3.2, and Ca_v_3.3 [71,72]. Although easier to differentiate at the molecular level or with genetic tools, pharmacological modulation of T-channels has been difficult due to the lack of selective blocking agents. Over the last couple of decades, we have been studying a library of neuroactive steroids with T-channel blocking and/or GABA-modulatory properties that could be promising novel general anesthetics.

**Figure 1:**
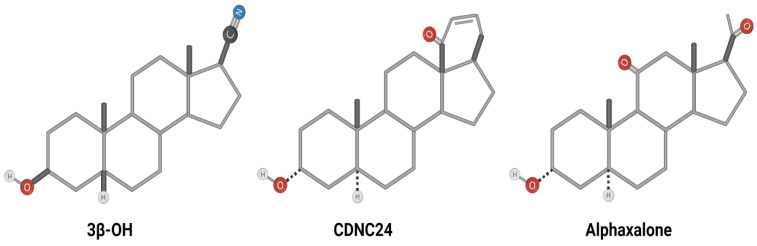
Synthesized neuroactive steroids—chemical structures. Chemical structures of synthesized steroids used in our studies: **3β-OH**—(3β,5β,17β)-3-hydroxyandrostane-17-carbonitrile; **CDNC24**—(3α,5α)-3-hydroxy-13,24-cyclo-18,21-dinorchol-22-en-24-ol; **alphaxalone**—5α-pregnan-3α-ol-11,20-dione.

Using in vitro and in vivo approaches, we have examined several synthesized neuroactive steroid analogues that could be considered effective hypnotics and potent analgesics while lacking neurotoxic effects in very young animals described with all other clinically used general anesthetics when administered at postnatal day 7. Three promising analogues we have identified and studied over the past decade are:

- **3β-OH** ((3β,5β,17β)-3-hydroxyandrostane-17-carbonitrile);
- **CDNC24** ((3α,5α)-3-hydroxy-13,24-cyclo-18,21-dinorchol-22-en-24-ol); and
- **Alphaxalone** (5α-pregnan-3α-ol-11,20-dione) (Figure 1).

### 3.2. Lack of Neurotoxicity after Prolonged Exposure Following Sufficient Depth of Hypnosis

Using the loss of righting reflex (LORR) as a behavioral correlate of hypnosis in rodents, we determined that 3β-OH, CDNC24, and alphaxalone were potent hypnotics (in a dose-dependent manner) and, importantly, when compared to the commonly used general anesthetics propofol and ketamine, all three were found to be safer based on calculated therapeutic indices [34,35] when administered to 7-day-old rat pups. For example, therapeutic indices for 3β-OH, CDNC24, and alphaxalone were 20, >88.23, and 31.41, respectively, whereas therapeutic indices for clinically used the general anesthetics propofol and ketamine were around 23. In spite of the fact that a couple of limitations dissolving these very hydrophobic compounds exist (i.e., maximum concentration ~2 mg/mL of CDNC24, whereas for alphaxalone it was up to 12 mg/mL in 25% cyclodextrin), it is worth mentioning no mortality was observed with CDNC24 at the highest dose we were able to administer, indicative of a very high therapeutic index (88.23).

Encouraged by their hypnotic potential and promise for being viable alternatives to currently used general anesthetics, we set out to examine their neurotoxic potential when administered during critical stages of brain development, especially for prolonged time periods. We compared their potential to induce significant developmental neuroapoptosis to the currently used general anesthetics propofol and ketamine while focusing on several brain regions: the cortex, thalamus, and hippocampus. Looking at caspase-3 activation, a common histomorphological marker of developmental neuroapoptosis, we found that neither CDNC24 nor alphaxalone anesthesia, when administered for six hours at equipotent doses to propofol, resulted in significant upregulation of neuroapoptosis compared to age-matched vehicle controls. On the other hand, propofol caused a significant and widespread upregulation of caspase-3 activity when compared to controls and CDNC24 or alphaxalone [35]. This very obvious increase in cell death after propofol exposure was observed to a similar extent among the aforementioned brain regions in comparison with controls.

Further comparison of neurotoxic potential of commonly used general anesthetics and novel steroid analogues was performed using equipotent doses of ketamine or 3β-OH where the findings were similar. Unlike ketamine anesthesia, which caused a more than 100-fold increase in caspase-3 activation compared to vehicle controls, a 12- hour 3β-OH anesthesia using the equipotent doses did not result in a significant increase in neuroapoptosis. The absence of caspase-3 activation after exposure to this neuroactive steroid, as well as the extent of the damage caused by ketamine, was consistent across all assessed brain regions. Jevtovic-Todorovic and associates have previously described that the aforementioned brain regions have very similar age-dependent sensitivity to the application of commonly used anesthetics [3]. Having said that, a unique period of vulnerability and abundant activation of neuroapoptosis in those regions is very severe beginning at the age P1 and P3, with the highest peak around P7 corresponding to the peak synaptogenesis, while the rapid decline of sensitivity to these agents is observed by the age P14 [3]. Therefore, we concluded that novel steroid analogues are effective hypnotics and are not damaging to the young brain at the peak of development across different brain regions. Together with our earlier work suggesting their potent analgesic properties [67,70,73], this led us to believe that they could be a promising line of new general anesthetics for use in a young population.

### 3.3. Lack of Long-Term Consequences in Cognitive Development

Before the histomorphological findings could be considered viable and potentially important, it was critical to examine whether there were any long-lasting behavioral impairments similar to what has been published with commonly used general anesthetics [8,9,10,11,12,13]. Although there is a long road ahead before we can establish the safety of steroid analogues in all aspects of behavioral outcomes reported with currently used general anesthetics [8,9,10,11,12,13,14,17,18,19], our first step was to focus on cognitive development, since the hippocampus, a brain region known to play an important role in learning and memory, is exquisitely sensitive to general-anesthesia-induced developmental neurotoxicity [9,10,11,12]. We used the eight-arm radial maze, a commonly used task to examine rodents’ spatial learning and memory, and were able to confirm that early exposure to 3β-OH during a peak of synaptogenesis did not result in long-term learning deficits in rats when assessed during adulthood. We confirmed that the commonly used general anesthetic ketamine caused a significant impairment in learning speed and ability to achieve a priori determined learning criterion, relative to control. In addition, the cumulative analysis revealed that ketamine-treated animals demonstrated a significant gap in learning abilities compared to 3β-OH [34]. Based on this evidence, we propose that neuroactive steroids may be a promising alternative to currently used general anesthetics when administered during the critical stages of brain development.

## 4. Novel Cellular Targets of Neuroactive Steroid Analogues Considered to Be Promising General Anesthetics

During a few decades of intense research, we have performed several structure–activity relationship studies looking at a variety of cellular targets of different neuroactive steroid analogues. Known to modulate neuronal activity in the peripheral and central nervous systems, the majority of synthesized neuroactive steroids have effects on neurosensory processing and neuronal excitability [65]. These effects are thought to be mediated primarily by actions at various ligand-gated ion channels, with much attention focused on the potentiation of currents mediated by postsynaptic GABA_A_ receptors [44,45,63,64,65]. Since most currently used GABA-modulatory general anesthetics share a neurotoxic potential, the initial focus was on identifying novel steroid analogues with the same analgesic and hypnotic properties, but with selective and potent blocking action on other cellular targets. The most attention has been focused on neuronal T-channels, with the hope that T-channel blocking analogues would be less neurotoxic. As stated earlier [47,74,75], we found that one such analogue is 3β-OH, a potent voltage-dependent blocker of T-currents in naive neurons (acutely dissociated dorsal root ganglion cells (IC50 at 3 µM)) [47] and in thalamic neurons from acute brain slices (IC50 at 2 µM) [76,77,78], without significant blocking effect on voltage-gated Na^+^ and K^+^ currents, N-type and L-type high-voltage-activated (HVA) VGCCs, or on recombinant GABA_A_ or NMDA-mediated currents [67,70,76,79]. To begin to understand the effects of the neuroactive steroid analogues of interest, we began with the studies of 3β-OH-induced effects on the average amplitude of calcium currents mediated by T-channels using acute brain slices from subiculum and ventrobasal thalamus [34]. Our analysis showed that 3β-OH decreased the average amplitude of the low-threshold Ca^2+^ spike in thalamic neurons while abolishing burst firing without significantly changing the resting membrane potential. It was noteworthy that these changes in firing were observed within the hypnotic brain concentrations, as confirmed in our pharmacokinetics experiments [34]. It was further determined that 3β-OH, unlike the endogenous neuroactive steroid allopregnanolone, was completely devoid of direct postsynaptic or extrasynaptic GABA-modulatory activity even at high concentrations. Furthermore, 3β-OH was reported to have an effect on the glutamatergic system by decreasing the probability of glutamate release from presynaptic terminals [34].

Of note, 3β-OH and allopregnanolone have been shown to inhibit rebound burst firing by decreasing T-currents in a voltage-dependent manner, while also suppressing long-term potentiation (LTP), an in vitro approach adopted for the studies of memory processes in the hippocampus. In addition, this finding was confirmed in our most recent study, which showed that the Ca_v_3.1 isoform was critical for thalamocortical excitability and oscillations, which may, at least in part, explain the hypnotic effects of 3β-OH [80]. Based on the work with 3β-OH, we concluded that non-GABA-modulatory cellular targets could be of interest for the development of safer general anesthetics.

An interesting twist to these conclusions was the latest observation of CDNC24 and alphaxalone, where both were found not to be neurotoxic to the young brain [35], despite having direct GABA-modulatory properties. How could this be reconciled? Is there some unique property of neurosteroid analogues that confer intrinsic ‘neuroprotection’ despite a GABA-modulatory effect? The initial experimental work suggests that in addition to postsynaptic potentiation of GABA_A_, both CDNC24 and alphaxalone presynaptically reduce spontaneous GABA release at hypnotically relevant brain concentrations [35]. Therefore, the lack of neurotoxicity of these steroids may be linked to minimization of excessive postsynaptic and extrasynaptic GABA_A_ activation, knowing that these receptors are excitatory at this stage of development (at postnatal day 7), contrary to the adult brain, as stated previously [66,67]. Thus, it is assumed that they are effectively balancing excessive postsynaptic and extrasynaptic GABA_A_ activation through modulation of α4 and/or δ subunits in extrasynaptic GABA receptors known to play an important role in general-anesthesia-induced hypnosis [81]. Propofol, on the other hand, was shown to have primarily postsynaptic effects resulting in upregulation of tonic GABA_A_ currents, as shown by an increase in spontaneous inhibitory postsynaptic current (sIPSC) decay time without a change in the event frequency [35]. Another interesting possibility that should be considered as a mechanistic explanation, at least in the case of alphaxalone, is its simultaneous blockade of T-channels (and possibly other voltage-gated calcium channels) and reduction of presynaptic GABA release in the synaptic cleft that may explain its hypnotic action while lacking the neurotoxic effects. Yet, we still do not have a precise explanation for pre- and extrasynaptic events mediated by these steroids.

**Figure 2:**
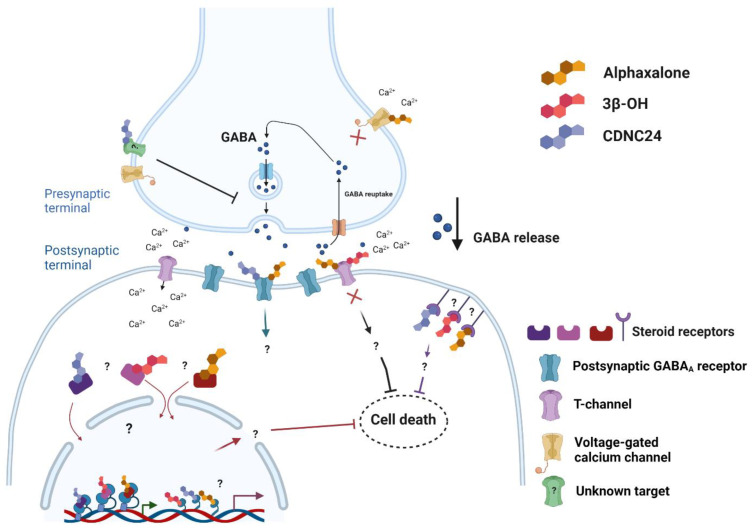
Summary of known and potential mechanisms of neuroactive steroids. 3β-OH inhibits postsynaptic T-channels without having a direct effect on postsynaptic GABA_A_ receptors. In addition to postsynaptic potentiation of GABA_A_, both CDNC24 and alphaxalone presynaptically reduce spontaneous GABA release, thus decreasing GABA content in the synaptic cleft. In addition to its effect on postsynaptic GABA_A_ receptors and reduction in presynaptic GABA release in the synaptic cleft, alphaxalone also simultaneously blocks postsynaptic T-channels. Additionally, based on the well-known abilities of steroid compounds to pass cellular membranes, there is a possibility that the explanation for the absence of cell death in neurons lies in their direct or indirect interactions with membrane-bound, intracellular, and nuclear receptors, either as agonists or antagonists. These targets, as well other potential targets on the presynaptic terminal, remain unknown.

Another possible explanation could be that their pharmacological effects are not only mediated via the cellular membrane receptors, but also via the nuclear receptors. For example, the work by Serrao et al. (2020) suggests that both allopregnanolone and alphaxalone activate pregnane-x-receptor (PXR) [82], a ligand-activated nuclear receptor that controls inducible expression of biotransformation enzymes and drug transporters [83,84]. Importantly, PXR is known to cross-talk with other nuclear receptors, thus controlling coactivation and activity of many transcription factors [83,84]. Hence, it is possible that the effects of 3β-OH, CDNC24, and alphaxalone we have discussed herein are not only mediated through the cellular membrane receptors, but are also the result of a complex interplay with the nuclear receptors (Figure 2).

At present, a causative link between electrophysiological and behavioral impairments and potentiation of GABAergic neurotransmitter systems has not been confirmed. It would be difficult to establish such a link, considering the fairly promiscuous nature of currently available general anesthetics, which are known to modulate other cellular targets (e.g., leak potassium channels, NMDA/AMPA subtypes of glutamate receptors, and glutamate excitatory amino-acid transporters (EAAT)-1/2/3) [1,27,31,85,86]. A unifying property of general anesthetics is their ability to silence neuronal activity in certain brain circuitries, thus hampering communication between various neuronal groups, a necessary component of achieving a hypnotic state [31]. Since neuronal communication and activity are crucially important for all aspects of developmental synaptogenesis (e.g., migration, differentiation, maturation, and dendritic arborization), it would be reasonable to propose that prolonged and unphysiological inhibition of neuronal activity and communication during general anesthesia, regardless of which cellular targets are being affected, would hamper timely and proper formation of neuronal circuitries crucial for cognitive development. In other words, the general inhibitory state, rather than any specific receptor or ion channel system, may be culpable. Our published work with neuroactive steroids challenges the validity of such speculation, and proposes the premise that steroid analogues are effective hypnotics that lack the neurotoxic potential observed with presently available general anesthetics.

## 5. Conclusions

Based on currently available information, we believe that novel neuroactive steroid analogues can be promising general anesthetics and effective analgesics. Importantly, they may be a safe alternative to currently used general anesthetics, since they provide hypnosis within the dose range that does not activate cell death pathways and enables normal cognitive development. These observations warrant further investigation of their long-term effects with an emphasis not only on learning and memory, but also on other aspects of behavioral development such as socioaffective growth, emotional maturation, and motor function. Inclusion of other mammalian species in addition to rodents would be prudent as well. Considering that many ‘safening’ strategies shown to alleviate the general-anesthesia-induced developmental neurotoxicity have not resulted in truly viable therapeutic alternatives, the need to focus on the development of new general anesthetics is long overdue. We owe it to our youngest patient population, particularly when prolonged and/or repeated exposures during critical stages of their brain development are a necessity that cannot be avoided.
